# A basic scientist’s reflections on research funding

**DOI:** 10.1186/s40697-015-0087-0

**Published:** 2015-12-01

**Authors:** Katalin Szaszi

**Affiliations:** Keenan Research Centre for Biomedical Science of the St. Michael’s Hospital, 209 Victoria Street Room 622, Toronto, ON M5B 1T8 Canada; Department of Surgery, University of Toronto, Toronto, ON Canada

Scientists are among the most enthusiastic people when it comes to talking about their work. Despite this, it seems that it is the funding system that Canadian scientists most often discuss these days, and not their findings and new ideas. Needless to say, research can only exist with good and secure funding, but when obtaining funding becomes a dominant part of investigators’ activity, the system has a problem. I am a cell biologist and physiologist recruited to Canada from Hungary. Generous funding helped me to complete my post-doctoral training here and to start my own lab a decade ago. The transition from post-doc was smoothened by one of the last Canadian Institutes of Health Research (CIHR) Senior Research Fellowships that gave 2 years of post-doctoral funding and 2 years of new investigator salary support. I was extremely lucky to have this opportunity, which is no longer available to current post-docs. However, as my independent work started to produce results, I had to realize how difficult it was to maintain even a modestly sized lab (one technician and two students) within a research institute. As the reality of inevitable rejections of first grant renewal applications set in, I was swept away by the struggle for funding and the never-ending cycles of reviewing. This experience, shared by many of us, shaped my views on the ills of the Canadian biomedical research funding system. I am sharing my thoughts on this important and complex topic, with the hope that it will be part of a fruitful discussion.

## The struggle for funding overtakes research

There is a general agreement that the funding system for biomedical research is broken. In fact, many researchers would describe our current situation as a deep crisis. Although we lack hard data to verify the darkening mood of the science community, we seem to be losing the creative, idea-centred environment required for leading-edge science. A common experience of many of us is a sense of hopelessness and bitterness due to the non-stop struggle for funding, and the lack of fairness and predictability in our professional lives. In fact, I have never before witnessed such desperation among my colleagues during my 17 years in research in this country.

As recently pointed out by the Canadian Society for Molecular Biosciences in a letter to the Minister of Industry, ‘the overwhelming majority of discovery research laboratories critically depend on the very competitive and most innovative open operating grant competitions’ [1]. As funding rates are falling, labs are underfunded, and the current funding environment threatens the existence of many labs across the country. Even well-established investigators, who have proven themselves excellent and productive scientists, are just one failed renewal application away from a crisis due to disruption in funding. High-quality research productivity no longer guarantees renewal of funding. ‘Publish or perish’ has been replaced by ‘publish AND perish’. As a result, we now spend less time focusing on ideas and research itself, and more time applying for funding. As I learned during my many cycles of resubmissions, enormous amount of resources and brain power goes into preparing grants, generating preliminary data and addressing often subjective requests from reviewers who might not even be in the committee to read the resubmission.

The cycles of resubmissions waste financial and human resources, but in addition to this, their detrimental effects on the psyche of all involved cannot be emphasized enough. I spent unimaginable number of hours rewriting the same grant for resubmissions, time I spent not doing research. Loss of time however was not the biggest problem. As many of us will testify, nothing reduces scientific creativity more than the increasingly desperate fight for funding, the inevitable soul-crushing rejections, and the constant pressure from competition. There is nothing less motivating than writing and reviewing grants, especially with the knowledge that the outcome more likely than not will be a rejection. The constant grant competitions with unpredictable outcomes are a major source of debilitating stress that crushes creative thinking. In addition, it is worth noting that science is a collaborative endeavour, where the rocky road towards discovery is best smoothened by sharing data instead of hiding them to protect one’s chances for funding. Overall, our funding system is turning scientists into entrepreneurs and managers, and forcing them into roles they have not trained for, never wanted as a career, and which requires a very different mindset than doing science. I was trained to be a scientist and Dragon’s den is not my world. Rules of the business world cannot be applied to science.

Training is also an important task of research labs. The funding crunch victimizes our trainees in more than one way. Students are facing a rapidly changing training environment, as fewer researchers can afford to train graduate students. Those labs that can still participate in training now rely too heavily on these inexperienced but cheap members of the research community to perform cutting-edge science. Absence in the labs of senior people, e.g. technicians and research associates who no longer can be paid from average grants, reduces the quality of both training and research, slows progress, and forces students into roles they often cannot fulfil. This is a fact well known to anyone who has tried to finish promising research projects with inexperienced students. Finally, who will be the next generation of researchers? Academia has lost its appeal and our struggles will hardly make our trainees want to follow in our footsteps.

## How did we get to this point?

There are two sides to this problem: stagnating funding, that has to support an increasing number of excellent labs, and a major shift in funding philosophy affecting existing labs. In the past decades, biomedical research yielded amazing results and the number of excellent research groups exploded in Canada. Unimaginable technical advances and the fast growth of the biotech industry that supports experimental research widened the scope and potential of research but also increased expenses. Despite these factors, in the past years, funding stagnated, leading to plummeting funding rates and a sharp fall in the value of individual grants. For example, the latest transitional operating grant competition of CIHR not only yielded an unprecedentedly low funding rate (below 15 %), but also saw a 27 % budget cut for every awarded grant. This hit especially hard, since in the past 2 years, we had only one competition per year instead of the previous two. The decrease in inflation-adjusted value of the Canadian Research and Development expenditures is well demonstrated by Chakma et al. who explored global trends in research funding during the period of 2007–2012 [2]. Their data show a negative growth in spending in Canada, the USA and Europe, which was in sharp contrast to the huge growth in Asia, most notably in China.

Along with stagnating funding, there is an ongoing shift in the funding philosophy, as demonstrated by the changing policies and funding priorities of CIHR and Natural Sciences and Engineering Research Council (NSERC). Especially problematic from my viewpoint is the rapidly decreasing appreciation of curiosity-driven fundamental research. The current trend rewards applied research and knowledge translation at the expense of basic science and highly rates projects with potential for commercialization, i.e. immediate ‘results’ and industry relevance. In contrast, projects that aim for a better understanding of fundamental mechanisms are increasingly out of favour. A second trend is that large portions of funding are directed towards selected research priorities, further reducing funding for investigator-initiated projects. As a result, applications that received lower ranking from review panels are funded based on their topic, with the danger that relevance takes precedent over quality. Yet another trend is that money is directed towards large labs and mega-projects, while starving many smaller labs.

These shifts that tilt the balance of funding are based on a few false beliefs. One such idea is that science is slow in benefitting society because academic researchers are reluctant to translate our knowledge. Accordingly, only an emphasis on translational research will stop scientists from doing ‘useless’ research for the sake of research itself and speed the application of discoveries to prevent and treat disease. However, the history of science tells a different story, as leading scientists continue to point out (e.g. [3–5]). In an excellent essay, David Botstein of Princeton University argues that great advances in biomedical science are the direct result of work by thousands of basic scientists whose primary goal was to understand fundamental mechanisms of biology [4]. It cannot be emphasized enough that basic science generates the discoveries that form the basis for translation. Thus, basic and translational science are complementary. As a powerful editorial in the journal Infection and Immunity puts it, ‘support for translational research must be accompanied by a robust investment in basic science, which provides the essential raw material for translation and continues to represent humanity’s best hope to meet a wide range of public health challenges’ [5]. Is it possible that the already accumulated knowledge is adequate to solve all diseases if we put more effort in translating? When considering this question, we must remember that knowing facts does not equal a deep and adequate understanding of mechanisms, something that is a prerequisite for successful translation. Thus, in the absence of good understanding of many processes in nature, the answer to this question is no. Despite this, applications aiming at mechanistic understanding of important processes with no immediate disease relevance stand little chance of funding.

A second false belief is that major steps can only come from large projects, while smaller groups are useless and not worth supporting. The truth is that discoveries can come from anywhere, and only a broad base of well-funded researchers can provide the environment and culture that will promote the creativity of and innovation by all participants. Interestingly, the need of a wide base of participants is well accepted in sports, where politicians and the public agree that we must support a large population of talents so some of them can reach the podium. This requirement is true for science too, with the important difference that in research, there are no winners and losers. Science does not move forward through large breakthroughs generated by isolated research groups (winners reaching the podium). Instead, main steps are due to increments provided by years and years of painstaking, slow work by many groups and slowly accumulating knowledge achieved through a multitude of failures [6].

## What about peer review?

Peer review was implemented to assure quality control and fairness. Contrary to its intended role, our current system is full of bias and greatly opposes risk taking and outside-the-box thinking, major attributes of leading-edge science. The problems of peer review were discussed in detail by Wheeldon and Gordon [7]. The ultimate goal of review is to select good projects with a high likelihood of success. But there is an inherent contradiction: due to the nature of scientific discovery, there is no good way of predicting which idea will work, which hypothesis will prove correct. Contrary, the more novel an idea is, the higher the chance that peer review will reject it as too risky.

Since funding decisions must be made; peer review increasingly relies on ‘objective measures’ including preliminary data and metrics to predict success. The requirement to support all aspects of a proposal by preliminary data is out of control. Significant portions of existing grants are now spent on generating data for the next grant. Due to the low funding rates, a large percentage of these data will never be further extended and published. The cycle of writing and reviewing mostly rejected applications leads to ballooning costs, money spent on nothing. Gordon and Poulin convincingly demonstrated how wasteful the peer review system is [8]. Using data from 2007, they estimated that the costs of preparing an NSERC discovery grant and its rejection by peer review ($40,000) exceeded that of giving every qualified investigator directly an average baseline discovery grant of $30,000. The amount of an average grant is usually higher at other agencies, but the requirements for expensive preliminary data are also more substantial.

As a consequence of falling success rates, the system is clearly overwhelmed by the increasing number of applications, a large proportion of which are excellent and would deserve funding. Reviewers are facing an almost impossible task of selecting the few winners from the large pool of excellent grants. They are often asked to assess projects in which they are not content experts, further increasing error.

To do the job, we are becoming overly critical, picky and unreasonable. Overall, reviewing is becoming an increasingly impossible and stressful job that unsurprisingly quickly leads to burnout. For applicants, lack of quality control and transparency in the review process is a huge issue. There is no mechanism to protest against mistakes and there is no accountability in the system. Addressing issues raised by reviewers does not guarantee a better ranking for the resubmitted grant, leading to endless cycles of resubmissions.

Since prior productivity is viewed as a good indicator for future success, much emphasis is put on this aspect when assessing applicants. For this, committees increasingly rely on metrics, but the use of these is problematic. This is a larger topic that I cannot address here in detail, so I will only mention one example: journal impact factors were not designed for the purpose of assessing the quality of applicants’ papers. The Declaration on Research Assessments (DORA) that was endorsed by a multitude of leading scientists and scientific organizations intends to reduce biases and inaccuracies when evaluating research by halting the practice of correlating the journal impact factor to the merits of a specific scientist’s contributions [9].

## What are the solutions?

There are no obvious, easy solutions for such a complex issue as improving the funding system. CIHR is currently undergoing a major overhaul, and other agencies, including the Kidney Foundation of Canada, are also changing their application process to improve peer review. It is too early to know the effects of these changes. An honest assessment and a better conversation between agencies and the research community will be vital. Importantly, improving the system will require not only more money, but also a rethinking of funding philosophy and peer review. Reform should aim at reducing the counterproductive struggle for funding. Increased funding security for some well-established researchers through the new CIHR foundation scheme is a good step. But with its long evaluation times and low funding rates, it leaves many outstanding applicants in an unfair funding limbo for extended periods of time. Without better funding for the project scheme, the system will also leave a large portion of labs unfunded. The balance also appears to be tilting further towards translation at the expense of discovery research, a trend that will ultimately be detrimental for innovation. Finally, eliminating face-to-face meetings will save money, but will also remove an important quality control step and thus will reduce fairness.

One possible solution could be to decrease the reliance of researchers on the grant system. Support provided by institutions could increase funding security. Quality control can be maintained by the careful selection process through which institutes hire the best researchers. Funding researchers and not specific projects can promote risk taking.

In summary, supporting a broad variety of research including basic and translational projects and a large cohort of committed researchers is of utmost importance and is the best way to generate an environment of innovation and translation, something that will surely benefit all Canadians.

## References

[1] Biosciences CSfM (2015). Letter to Honourable James Moore.

[2] Chakma J, Sun GH, Steinberg JD, Sammut SM, Jagsi R (2014). Asia’s ascent—global trends in biomedical R&D expenditures. N Engl J Med.

[3] Collins FS (2012). NIH basics. Science.

[4] Botstein D (2012). Why we need more basic biology research, not less. Mol Biol Cell.

[5] Fang FC, Casadevall A (2010). Lost in translation—basic science in the era of translational research. Infect Immun.

[6] Hocking W (2011). In praise of incremental steps and modest ideas. Physics Canada.

[7] Wheeldon J, Gordon R (2011). The innovation gap in Canadian academic research.

[8] Gordon R, Poulin BJ (2009). Cost of the NSERC Science Grant Peer Review System exceeds the cost of giving every qualified researcher a baseline grant. Account Res.

[9] Alberts B (2013). Impact factor distortions. Science.

